# HIV Rapid Testing in the General Population and the Usefulness of PrEP in Ecuador: A Cost–Utility Analysis

**DOI:** 10.3389/fpubh.2022.884313

**Published:** 2022-06-17

**Authors:** Paulina Quirola-Amores, Pablo Espinosa, Sebastian Oleas, Isabel Hernandez, Aquiles R. Henriquez, Enrique Teran

**Affiliations:** ^1^Instituto de Microbiología, Universidad San Francisco de Quito, Quito, Ecuador; ^2^Colegio de Ciencias de la Salud, Universidad San Francisco de Quito, Quito, Ecuador; ^3^Facultad de Medicina, Medicina, Universidad de Las Américas, Quito, Ecuador; ^4^Instituto de Economía, Universidad San Francisco de Quito, Quito, Ecuador; ^5^One Health Research Group, Universidad de Las Americas, Quito, Ecuador

**Keywords:** HIV screening, HIV treatment, ART, PrEP implementation, DALYs, cost–utility analysis

## Abstract

**Introduction:**

HIV is considered one of the most important chronic transmitted diseases worldwide. The Joint United Nations Program on HIV/AIDS in 2020 proposed the strategy “95–95–95” which goals to achieve a 95% of cases identified, receives ART, and will have achieved suppression of the virus. In Ecuador by 2020, according to the Ministry of Public Health, 45,056 persons are living with HIV, principally men between 15 and 49 years, and a mortality rate of 4.8/100,000 habitats. This study aims to determine the cost–utility of applying an early screening to a sexually active population vs. only a high-risk population and if the use of PrEP is justified depending on different contexts.

**Methods:**

For the cost–utility evaluation, it was compared: (a) HIV screening performed only in the high-risk population vs. HIV screening in all population sexually active; and (b) the use of ART only for HIV treatment vs. ART as a treatment in diagnosed cases and the use of PrEP (only at a high-risk population of acquiring HIV). Calculation and weight of DALYs for HIV/SIDA were obtained through WHO guidelines. To generate the Markov model for HIV/AIDS, subjects were classified as symptomatic or asymptomatic, as well as the HIV deaths.

**Results:**

Cost–benefit analysis (CUA) showed that ICER for early diagnosis had a negative value which means a saving if the strategy will be implemented as a regular test (–$591, –$4,360) and −108 and −934 DALYs, in the case of ART and PrEP, ICER the $30,541–$59,410, which resulted in more than the GDP's threshold and health years between 2,511 and 10,635 in the general population. With a reduction of 70% in the assigned budget for the early diagnosis, Ecuadorian people could lose between 4 and 6 DALYs, while if the budget reduces more than 50% to ART, it will generate a loss of 10–12 years of healthy life.

**Conclusion:**

CUA demonstrates that an early diagnosis in a sexually active population is cost-beneficial. This, combined with ART or PrEP, is ideal to add years of healthy life.

## Introduction

HIV is considered one of the most important chronic transmitted diseases worldwide. Many cases have been reported since its appearance. In 2020, there were 37.7 million (30.2–45.1 million) people living with HIV, and 1.5 million (1.0–2.0 million) new cases emerge every year worldwide (1). A couple of decades ago, the chances of surviving more than 10 years with HIV were slim. Today, a considerable decrease in HIV mortality was experienced thanks to antiretroviral therapy (ART) (2). The UNAIDS (the Joint United Nations Program on HIV/AIDS) in 2020 proposed the strategy knowing how “95–95–95” which goals to achieve by 2030 a 95% of cases identified, receives ART, and viral loads undetectable worldwide (3). Governments invest considerable amounts of their capital to prevent and control HIV, mainly for early detection by rapid screening, education, and treatment. Even though great results have been reached with these approaches, many undiagnosed cases are responsible for a continue transmission. The US Centers for Disease Control and Prevention since 2006 has recommended screening to all subjects between the ages of 13–64 because there is a trend to overlook some cases due to the misconception of only testing high-risk populations.

Consequently, undiagnosed patients have not received ART, and an increase in morbimortality has been observed (4). Other factors associated with this phenomenon have been identified (especially in low-middle income countries), like loss of clinical follow-up, treatment withdrawal, and rising resistance to some drugs. Also, a lack of adequate data collection tools makes HIV control a real challenge, suggesting that we will not probably reach the estimated goals soon (5). Thus, reinforcement of prevention and correct use of public health resources is necessary to optimize control strategy expenses. A constant evaluation of obtained results in different contexts is the cornerstones of effective management.

In Ecuador for 2020, according to the Ministry of Public Health (MSP), 45,056 persons are living with HIV, principally men between 15 and 49 years, and a mortality rate of 4.8/100,000 habitats (6). The implementation of basic worldwide recommendations is mainly focused on getting free access to ART. An opportune clinical management-led 79% of HIV carriers known their status, 73% were under ART, and 82% possessed undetectable viral loads in the last few decades (7). Undoubtedly, those results are encouraging and reflect a good approximation. Albeit, a considerable percentage of asymptomatic cases are not identified each year, basically because of the pathophysiology of the disease, its development, and an accurate screening on potential transmitters of HIV infection (8).

Cost–utility studies have been conducted for a long time. They are useful tools to decide, with evidence-based criteria, the best strategy/treatment to scale up in different contexts according to the health system's capacity. Studies usually use local or international data like prevalence, incidence, rates, loss of health, poverty, and economic and social determinants. The cost–benefit analysis by “top-down” considers general spending in a particular event and then calculates the cost (based on the potential use, time invested, personal, etc.), while the “bottom-up” manages a specific event considering their previous use (evidence and records) in different settings and unitary estimate costs (9). Usually, both are used in a mixed way, especially in low-middle income countries (LMIC), to create indexes and ratios and establish whether an intervention is a viable option, according to each local health assigned budget and the potential benefit to a specific population.

One of the most important is the incremental cost-effectiveness ratio (ICER), where variables like cost of each analyzed strategy denote if programs are economically efficient with the possibility to adapt according to each reality, and measurements of health improvement associated (like clinical outcomes and increasing of survival) through quality-adjusted life expectancy (QALY) that is the time spent in one state of health, in an attempt to reflected community perception (10). Disability-adjusted years (DALYs) is a measure that points out the amount of life in years lost due to unexpected death and the years of productive life lost due to disability (according to WHO) and estimates the benefit in health for a determinate population (11).

In a report from 2012, Ecuador mentions that the HIV government strategy allocates at least 50% of the annual budget only in prevention and ART (8). Still, it is not well understood, which are the criteria for this allocation of money. When national authorities decide that it is time to change or modify the HIV control policies, what are the parameters, results, and surveillance made for a clear assessment of capacity implementation? The recommendation of massive testing in subjects between 13 and 64 years was established by the Centers for Disease Control and Prevention (CDC) in 2006, without considering any risk factor (47), but since 2019 Ecuador made official this statement to the active sexual population and is pending how to perform it and measure the results of this intervention, calculate the spending, and corroborate if the suggested guidance is appropriate to our national reality.

On the contrary, pre-exposure prophylaxis (PrEP) has gained the field in clinical practice how prevention mechanism, especially at a high-risk population with different sexual behaviors with a proven beneficial effect, is still considered very expensive. An early HIV diagnosis has been demonstrated to reduce the emergence of new cases; however, some troubles have been experienced related to the adherence and follow-up once prophylaxis was initiated. This study aims to determine the cost–utility of applying an early screening to a sexually active population vs. only a high-risk population and if the use of PrEP is justified depending on different contexts (12).

## Methods

This study does not include human subjects or personal data; therefore, it did not require review or approval by an Independent Review Board (IRB). Data collected for this analysis were obtained between January and December 2020. The study was conducted in two groups: (1) people between 10 and 72 years at high risk of HIV infection (sex workers, GLBTI community, or pregnant women); and (2) general population between 10 and 72 years with a standard risk to acquire HIV. Also, for the cost–utility evaluation, it was compared: (a) HIV screening performed only in the high-risk population vs. HIV screening in all sexually active population; and (b) the use of ART alone for HIV-positive subjects vs. ART as a treatment in diagnosed cases plus the use of PrEP (only at a high-risk population of acquiring HIV).

Information was obtained from the HIV/AIDS National Bulletin of Ecuadorian Ministry of Public Health (13), while socio-demographic and health status variables required for the Markov model (14) were obtained from the National Health and Nutrition Survey (15), the Global AIDS Monitoring Report from Ecuador (16), other general info from the National Institute of Statistics and Censuses—INEC (17). ART/PrEP usage data were from the projection of the Pan-American Health Organization (18).

The Ministry of Public Health of Ecuador receives its budget for to each strategy or program (in this case, HIV/AIDS). It is calculated according to National resources/incomes, this information was extracted from the Global AIDS Monitoring (GAM), and some costs like budget handled in local prevention were obtained from PAHO reports of 2017, from the National costs list for the provision of services MSP 2012 (19), and the 2019 National Clinical Guidelines (48).

Calculation and weight of DALYs for HIV/SIDA were obtained through WHO reports (20), from the study of Murray et al. (21), and for the probabilities of change in health status (required in Markov's model), data from Haeussier et al., Sanni-Oba et al., and Vandewalle et al. were used (22–24).

**Figure 1:**
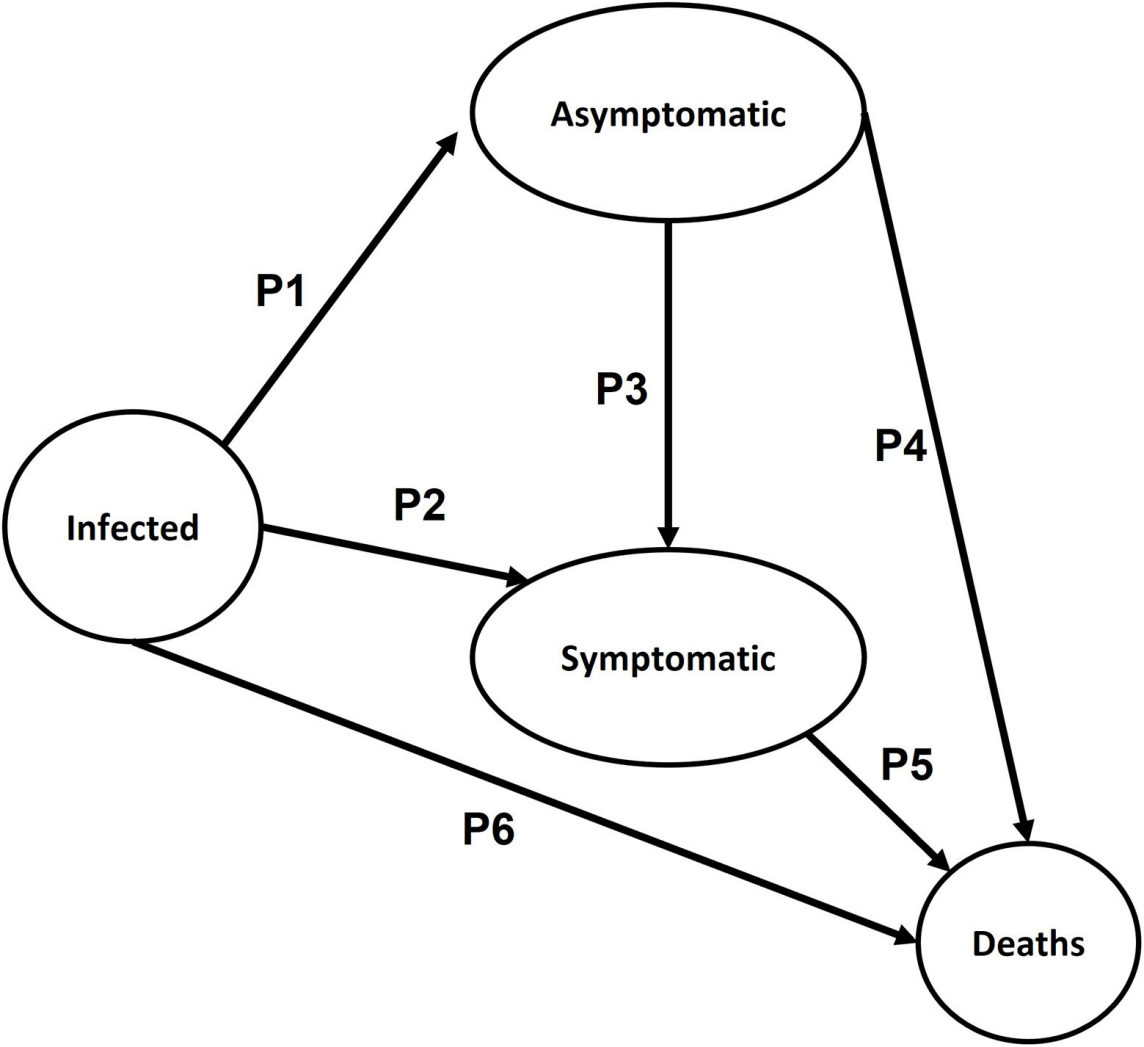
Diagram of Markov's Model for cost–utility analysis (CUA) for general/early HIV screening vs. only to high-risk population and the use of TAR/PrEP.

To generate the Markov model for HIV/AIDS, the analysis was based on *infected* subjects (*I*) who are HIV-1 carriers and know about their status. They were classified as *symptomatic* (*S*) or *asymptomatic* (*AS*), as well as the *HIV deaths*, both related and unrelated (Figure 1). It was also considered the probability of an infected subject being asymptomatic (*P1*), an infected subject to be symptomatic (*P2*), an asymptomatic to become symptomatic (*P3*), and the probabilities (*P4, P5*, and *P6*) that each state has for death (14).

To model each scenario, two types of data were employed: deterministic (based on values entered in Microsoft Excel V16.40) and stochastic (initial values generated randomly in the simulation cycles). Some adopted parameters were as follows: the probabilities of changing status (25), age disability weighting of DALYs and disability weights for HIV (WHO), baseline costs for HIV testing, PrEP, and ART (26).

To calculate the costs, it was used the proportion of screening tests performed (third and fourth generation) for the risk population (27), as well as the percentage of the national budget assigned for the purchase of ART in Ecuador (18). To estimate the variation that occurs with costs and DALYs in the simulated population, a matrix designed in Microsoft Excel was generated based on a previous design by Edlin et al. (14) (https://hta-modelling.leeds.ac.uk/downloads/) that recommends 100 cycles (simulations) for a better estimation of those values.

Models were generated with parameters for cost–utility estimation and considering both scenarios (the time of screening and the use of ART/PrEP). Then, it obtained the maximum and minimum cost for screening tests, ART/PrEP yields, and DALYs through the achieved simulations. All these variables were used in the probabilistic sensitivity analysis (PSA).

**Figure 2:**
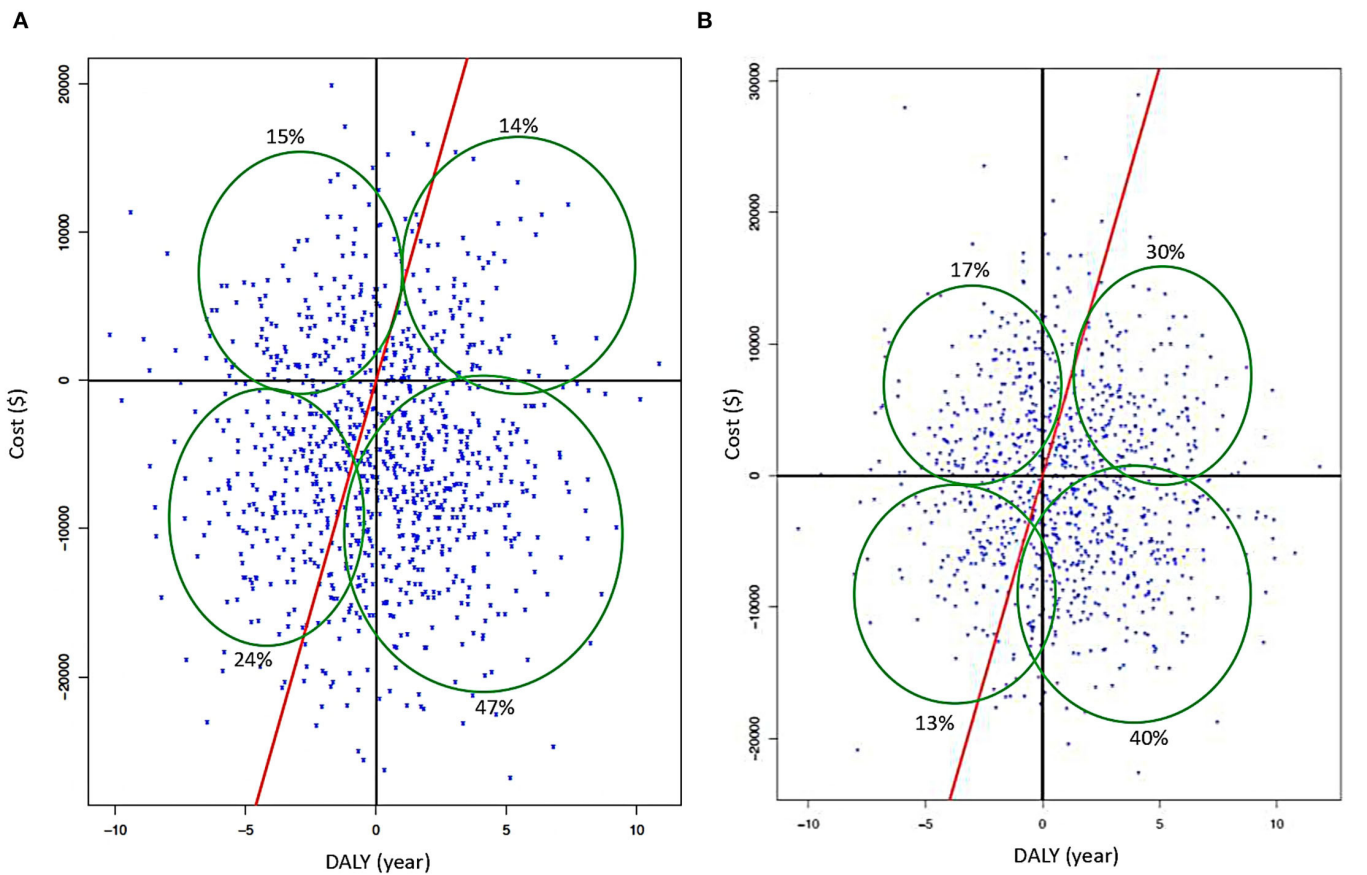
**(A)** Cartesian plane from PSA when an early screening is applied with deterministic data. **(B)** Results of PSA for early screening using stochastic data (red continuous line represents GDP's threshold; cost is in American dollars and DALYs in years).

**Figure 3:**
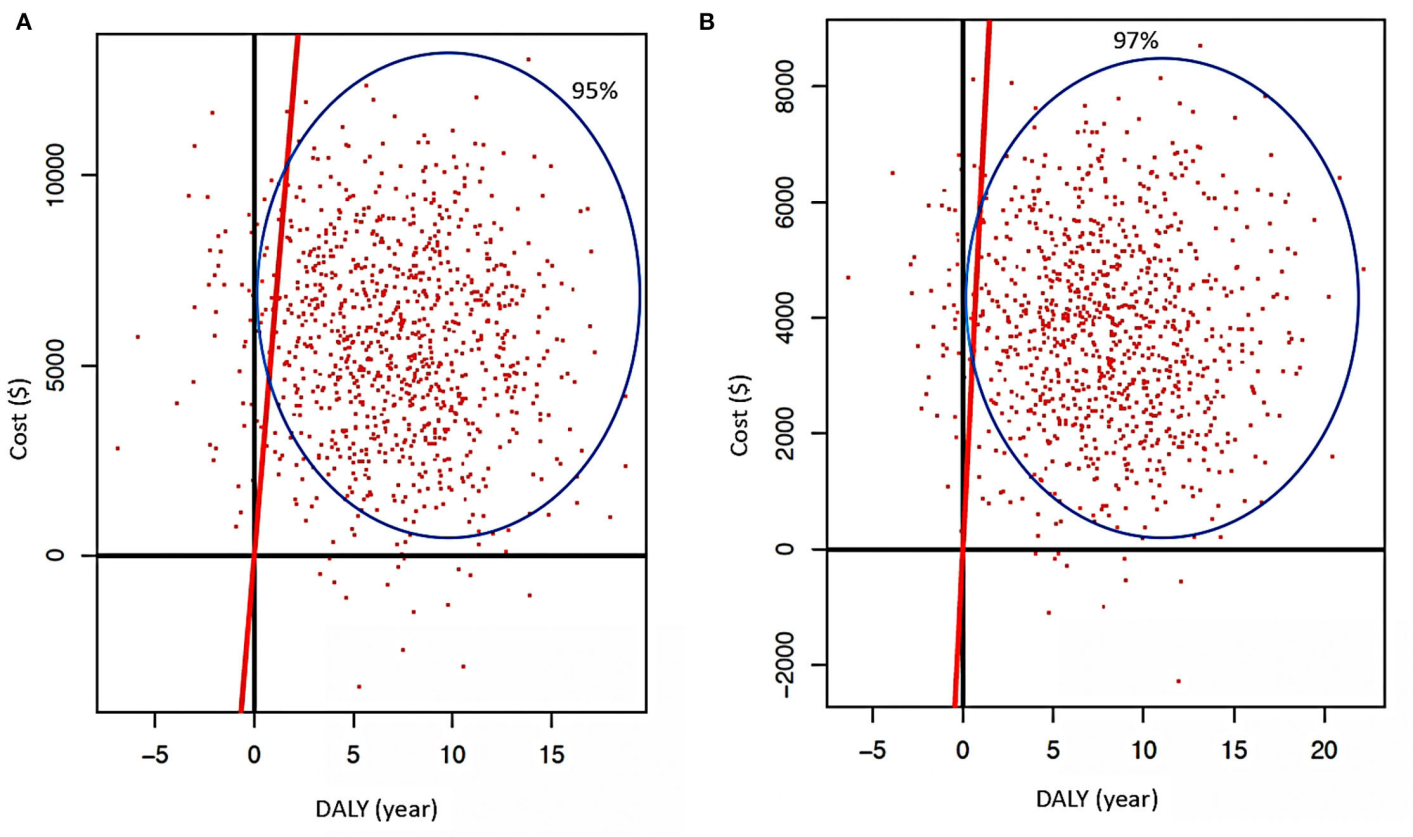
**(A)** Cartesian plane representing PSA when ART is used as treatment without other intervention (traditional) is applied with deterministic data. **(B)** Results of PSA when ART is used (regular) using stochastic data (red continuous line represents GDP's threshold; cost is in American dollars and DALYs in years).

With the above results, two PSA scenarios were generated using R Studio (V1.2.5042) based on the principles by Edlin et al. (14) and the Monte Carlo model for screening the following hypotheses: random gamma distribution (k = 3; θ = 0.001), DALYs with normal random distribution (μ = 3.46, σ^2^ = 3.36), and for ART/PrEP hypothesis costs. Hypotheses were considered as having a normal random distribution (k = 5, θ = 0.001) (Figure 2), and 3D graphics of both scenarios were generated through a script in MATLAB (V:R2020b) (Figure 3).

Then, there were incorporated the results obtained by Markov's Model (minimum and maximum costs of screening, ART/PrEP, and their corresponding DALYs), with an iteration of 1,000 cycles, obtaining cost–utility indexes: NHB (net health benefit that represents the impact on population health when a new intervention is introduced) (Equation 4), NBM (a net monetary benefit that represents the value of an intervention in financial terms when a willingness to pay is known) (Equation 3), and incremental cost-effectiveness ratio (ICER that is the economic value of an intervention, compared with an alternative (comparator) (Equation 5) (Figure 2).

For the cost–utility analysis (CUA), the PSA indexes were generated before being used to develop a Cartesian plane divided into quadrants, based on the model by Ho et al. (28), and a threshold (λ) according to the growth domestic product (GDP) in the year of corresponding analysis (here 2017), to determine whether the ICER is lower or higher than λ (29) in the two proposed scenarios. At the same time, we generated other indexes of cost–utility, that is, average cost of the strategy (CEM, Equation 1) and amount of health lost (HL, Equation 2) in DALYs years; to determine whether the cost of intervention reduces years of unhealthy life (this value is intended to be near to zero) (29). Later, we calculate the probability of density (which means adopting the cost–utility measurements affect the DALYs).


(1) $$\begin{array}{r} CEM=\frac{anual\;treatment\;cost}{anual\;treatment\;DALYs\;}\left[DALYs\right] \end{array}$$

(2) $$\begin{array}{r} Amount\;of\;health\;lost\;\left(HL\right)=\frac{NMB}{\lambda }\;\left[DALYs\right] \end{array}$$

(3) $$\begin{array}{r} Net\;Benefit\;Money\;\left(NBM\right)=CostA-CostB \end{array}$$

(4) $$\begin{array}{r} Net\;Health\;Benefit\;\left(NHB\right)=DALYsA-DALYsB\;\left[DALYs\right] \end{array}$$

(5) $$\begin{array}{r} Incremental\;Cost\;Effectiveness\;Ratio\;\left(ICER\right)=\frac{NBM}{NHB}\;\left[DALYs\right] \end{array}$$

## Results

In a scenario where all sexually active population is tested for HIV, simulations for CUA have a negative value for ICER, showing that investment between –USD 591 and –USD 4,360 could represent a future saving if this testing strategy is implemented. Screening for the high-risk population only generates a year's life between 108 and 934, which should be done to diminish one DALY year in the population with an early diagnosis. Both values are less than GDP's threshold (Table 1), and they are showing a correlation with the deterministic model, where is seven times less than the based threshold, and in the stochastic model, it was 0.095 times less than threshold.

**Table 1:** CUA results with deterministic and stochastic models applied to the different scenarios (early diagnosis and ART/PrEP) scenarios.

**Treatment/** **intervention**	**Annual cost ($)**	**Annual DALYs**	**CEM (USD)**	**ΔCost (USD)**	**ΔDALY**	**NHB (years)**	**NMB (USD)**	**λ (USD)**	**Health gained**	**ICER**
**Deterministic model**										
ART	71,673,908.3	9,245.31	7,752.45	−66,086,300	−2,163.795	66,086,299.6	2,163.795	6,213.5	−10,635.92	30,541.84
PrEP	5,587,608.75	7,081.51	789.04							
**Stochastic model**										
ART	19,364,060.5	8,133.177	2,380.87	−15,608,190	−262.716	15,608,189.9	262.716	6,213.5	−2,511.98	59,410.88
PrEP	3,755,870.54	7,870.461	477.21							
**Deterministic model**										
Late diagnostic	8,675,205.56	4,471.10	1,940.28	4,742,968.08	−108.63	−4,742,968.1	108.632	6,213.5	763.33	−43,660.87
Early diagnostic	13,418,173.6	4,362.47	3,075.82							
**Stochastic model**										
Late diagnostic	10,171,819.2	5,773.87	1,761.70	552,614.57	−934.36	−552,614.57	934.36	6,213.5	88.94	−591.43
Early diagnostic	10,724,433.7	4,839.50	2,216.02							

Considering ART and PrEP use, in both scenarios, the ICER showed that, if we increase the investment in these strategies (using them together), they could increase health in 2.51–10.6 in the simulated population. Even though the investment is high, it is under the cost-effectiveness threshold. In addition, the ICER demonstrated that for a decline of one DALYs, we need to invest among USD 30,541–USD 59,410 per year, resulting in more than the GDP's threshold (Table 1), exhibiting a relation with the deterministic model regarding, which is five times more than the threshold, and nine times more than the stochastic.

**Figure 4:**
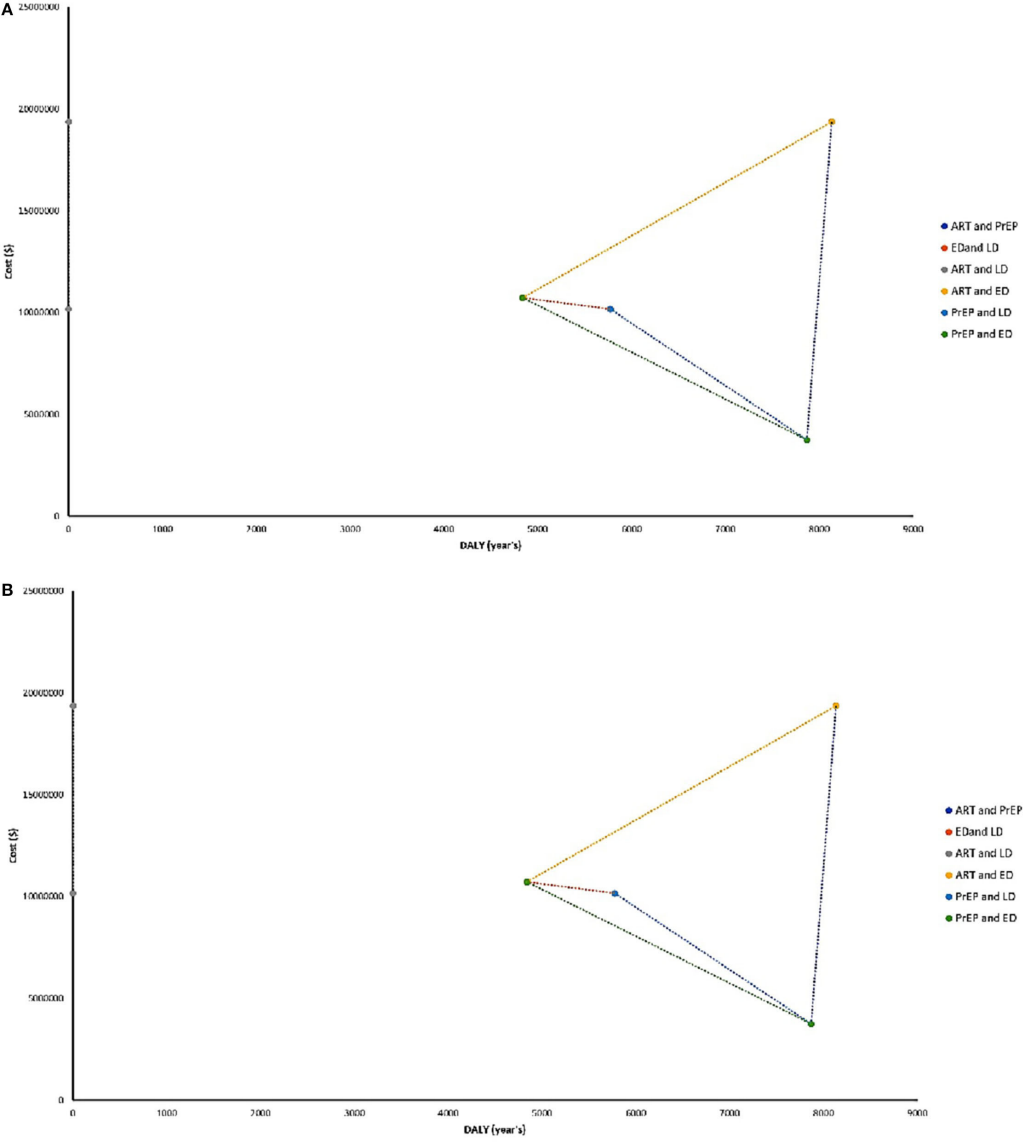
**(A)** ICERs with both analyses using a deterministic data distribution. **(B)** ICERs with both analyses using stochastic data distribution. ART, antiretroviral therapy; PrEP, prophylactic treatment; ED, early diagnosis; LD, late diagnosis.

Both the use of PrEP in a high-risk population and the early screening in the general population are more cost-beneficial than applying an early screening combined with ART in diagnostic patients as a strategy. In contrast, if ART is used only on diagnosed patients and PrEP in high-risk populations without an early screening, it would imply a reduction in costs but an increase in DALYs. Likewise, considering stochastic data, the use of PrEP at high-risk populations combined with an early screening at the general population is more cost-beneficial than the use of ART in diagnosed patients with an early screening from the general population. This combination of strategies showed that PrEP in a high-risk population with or without an early screening generates a lineal boost in DALYs. Finally, in the screening only to high-risk groups and the use of ART at infected subjects, higher economic spending will occur (exponential trend) and an increase in DALYs (Figures 4A,B).

The early screening (first scenario), using stochastic and deterministic data, was a high-cost strategy with an enormous benefit, as the majority of simulated data are under the GDP's threshold. In the stochastic data, more than 85% fit under the mentioned threshold (Figure 2A). If the traditional screening continues, data of stochastic and deterministic models showed a high-cost–utility and under the GDP's threshold. However, the stochastic data showed a better cost-effective trend (Figure 2B).

**Figure 5:**
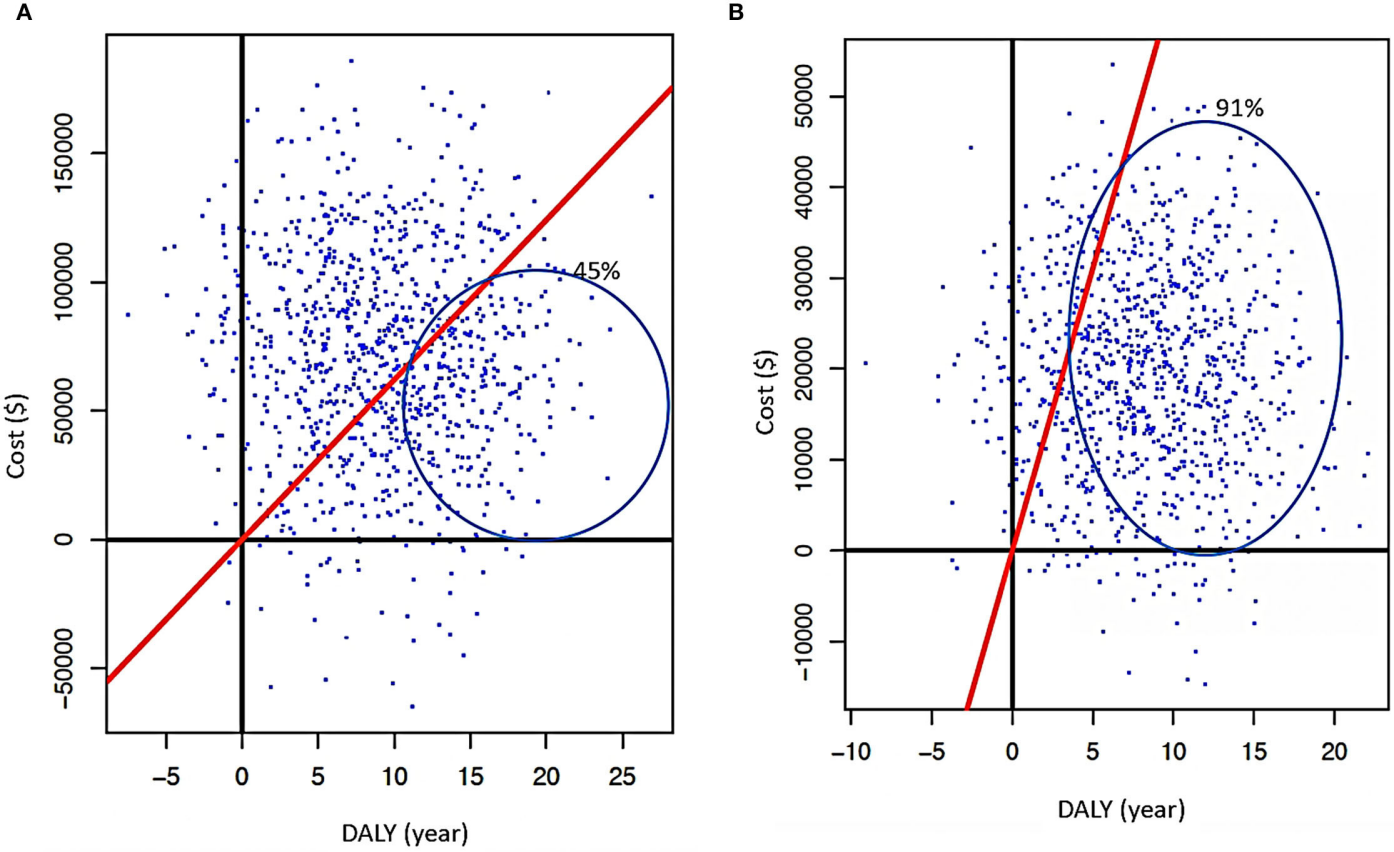
**(A)** Cartesian plane representing PSA when PrEP is used without other intervention is applied with deterministic data. **(B)** Results of PSA when PrEP is used (at high-risk populations) using stochastic data (red continuous line represents GDP's threshold; cost is in American dollars and DALYs in years).

For early diagnosis in both models, 60% of simulated data is under the GDP's threshold showing the strategies are highly beneficial, regarding low economic cost and loss of benefit lower than 30% for the combination of both (Figures 2A,B). When ART is employed like treatment (without other reinforcement strategies) while considering deterministic data, this strategy alone proves to be beneficial but represents a significant cost (finding 45% of simulated data under GDP's threshold). Considering only PrEP as the only approach at the high-risk population, simulations with stochastic and deterministic data demonstrate that the use of prophylaxis is beneficial but is extremely expensive (90% of these data are near the GDP's threshold), showing that the benefit could diminish if the cost increases (Figures 5A,B). When ART and PrEP are combined, 50% of stochastic and deterministic data were placed under GDP's threshold. As a result, both strategies are cost-beneficial when merged, representing an average economic expense, with a loss of benefit of at least 30% caused by their combination (Figures 3A,B).

### PSA for Payment Capacity and DALYs

**Figure 6:**
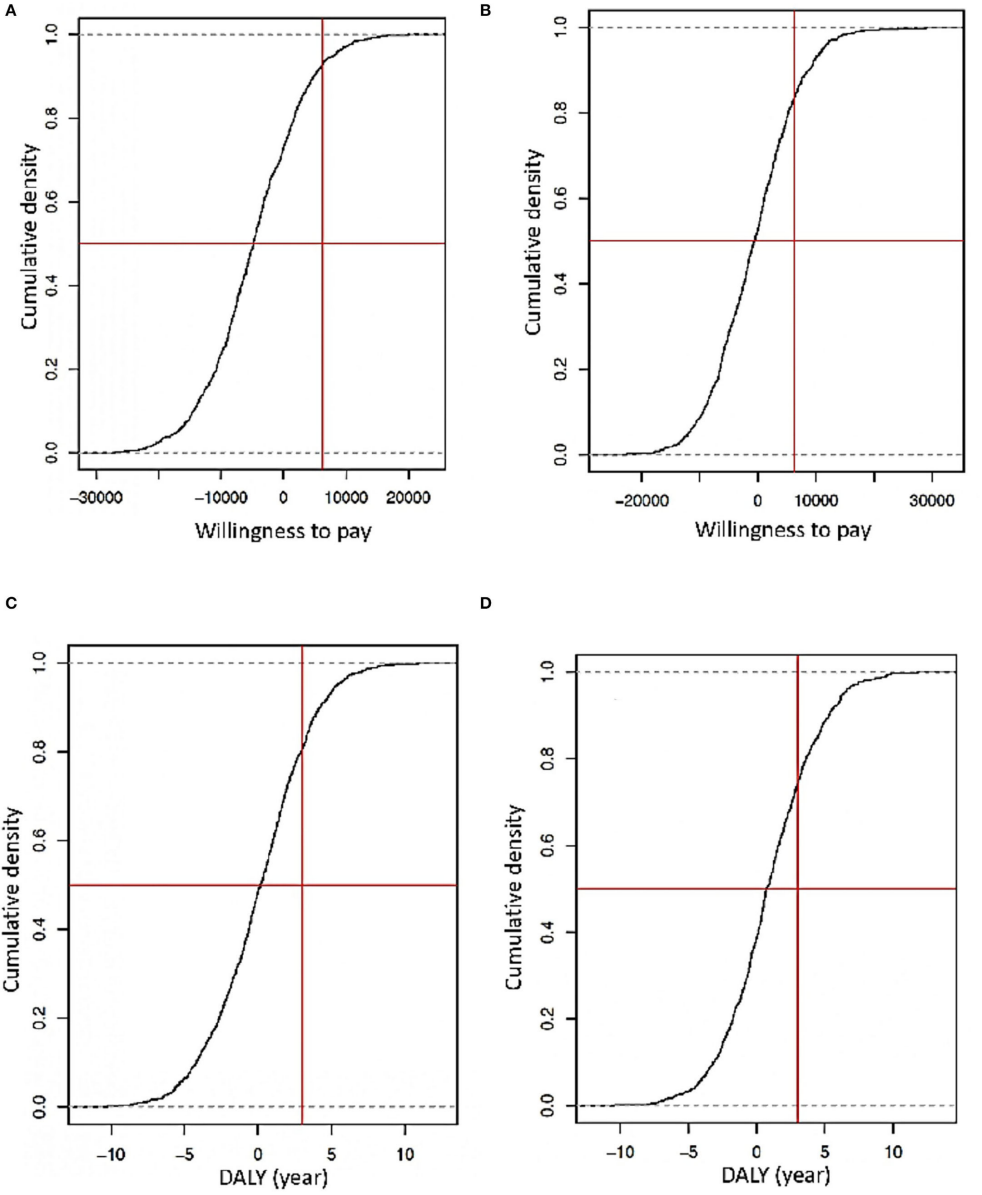
**(A)** Simulation of the capacity of willingness considering an early screening scenario with deterministic data diagnosis. **(B)** Simulation of the capacity of willingness considering an early screening scenario with stochastic data diagnosis (the Y is the probability that a spend is accepted how a part of an implemented strategy, X is the capacity of the willingness of an indeterminate government approach concerning GDP that is the vertical red line, and the horizontal red line the threshold of logistic regression of 50%). **(C)** Simulation of DALYs loss or gained using deterministic data diagnosis. **(D)** Simulations of DALY's years gained or loss using stochastic data diagnosis (the Y-axis is the probability to lose or gain years of life by the strategy. The red vertical line represents standard DALYs lost by a late diagnosis, and the horizontal red line is the threshold of logistic regression in 50%).

In an early screening scenario, a probabilistic sensibility analysis considering stochastic and deterministic data revealed that the investment for this intervention represents around 10,000 USD per person in the HIV/AIDS program per year. In contrast, with the regular screening and deterministic data, the investment would be 6,500 USD per person in the HIV/AIDS program per year (Figures 6A,B). Furthermore, considering the PSA DALYs with stochastic and deterministic data, in the hypothetical case that the National Authorities decide to decrease 50% of the budget that will be employed for early screening, there will be a loss of at least 5 years of healthy life. In the case of traditional screening with the same decreased percentage of the budget, the loss of years of health will be years of DALYs (Figures 6C,D).

Even more PSA simulations with stochastic and deterministic data indicate that the best cost-beneficial strategy of early screening includes general and high-risk populations. The investment to do this would be around 9,500 USD per person in the HIV/AIDS program per year. Without this strategy and a hypothetical reduction of 70% in the assigned budget, people could lose between 4 and 6 healthy years, but with the early screening of the general population, they could have 3–5 years of a healthy life, with a saving in resources of at least 20–30% (Figures 6A,B).

**Figure 7:**
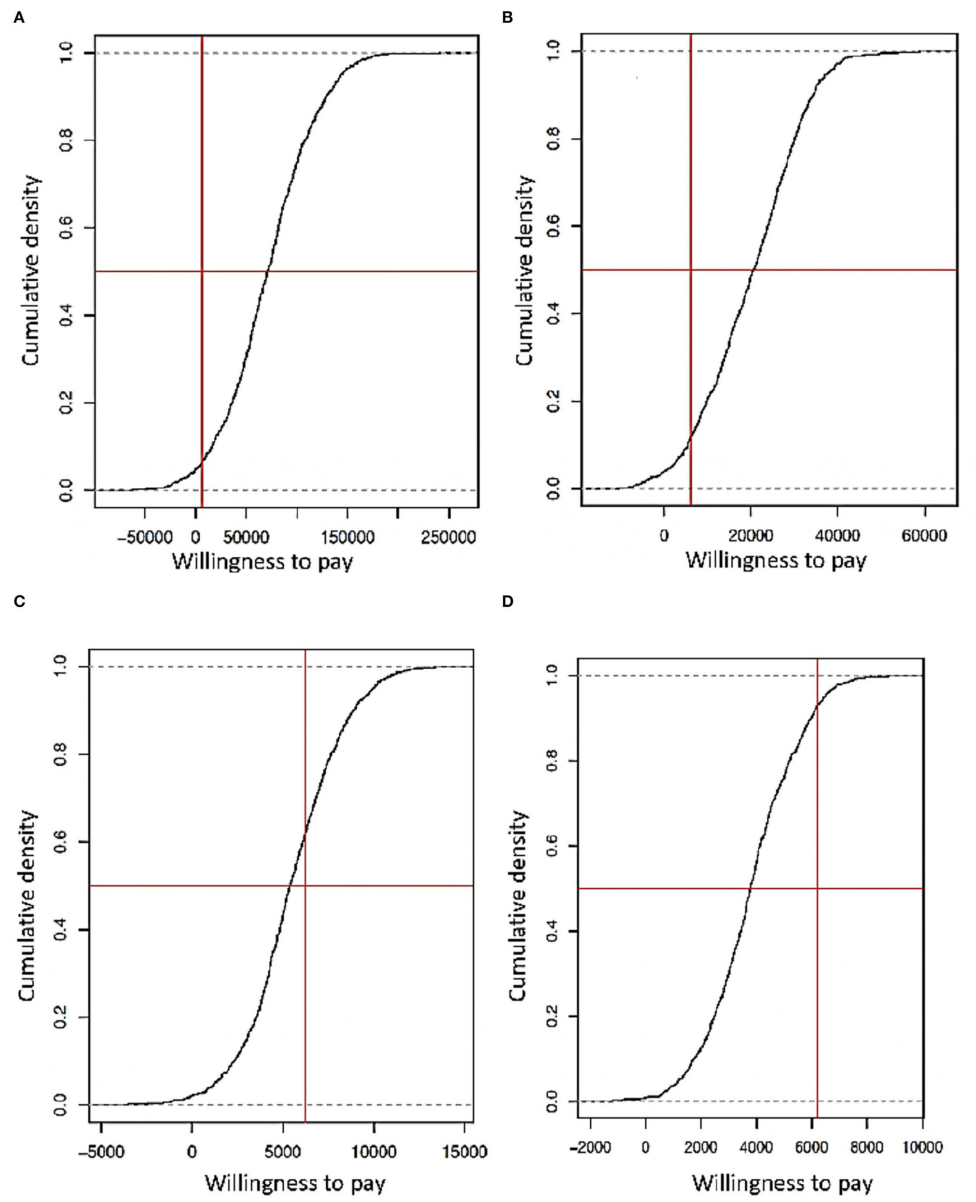
**(A)** Simulation of the capacity of willingness considering the use of only ART with deterministic data. **(B)** Simulation of power of willingness viewing the using singular ART with stochastic data. **(C)** Simulation of the capacity of willingness considering the use of PrEP like prevention with deterministic data. **(D)** Simulation of the capacity of willingness considering only PrEP like prevention with stochastic data (the Y-axis is the probability that an expending would be accepted and forming part of the implementation strategy, X-axis represents how much a government can spend on a strategy according to its GDP, represented by a vertical red line, and the horizontal red line is the logistic regression threshold of 50%).

**Figure 8:**
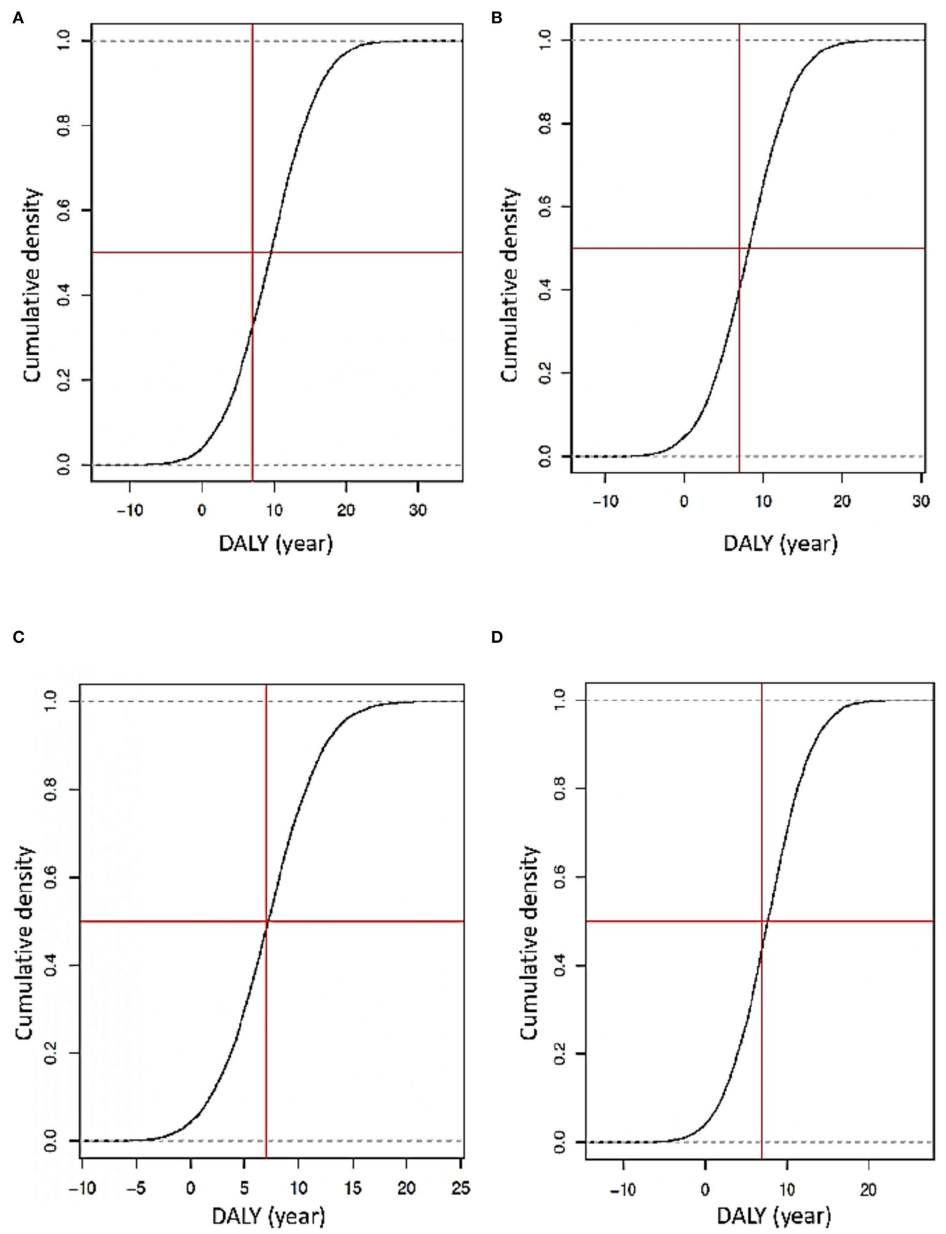
**(A)** Simulation of DALY's years' loss or gain using deterministic data for the exclusive use of ART. **(B)** Simulations of DALY's years gained or loss using stochastic data for ART use alone. **(C)** Simulations of DALY's years gained or loss using deterministic data for PrEP like prevention. **(D)** Simulations of DALY's years gained or loss using stochastic data for PrEP like prevention (the Y-axis is the probability of loss or gain years of life due to an implemented strategy. The vertical red line represents the standard DALYs lost by a later diagnosis, and the horizontal red line is the logistic regression threshold of 50%).

**Figure 9:**
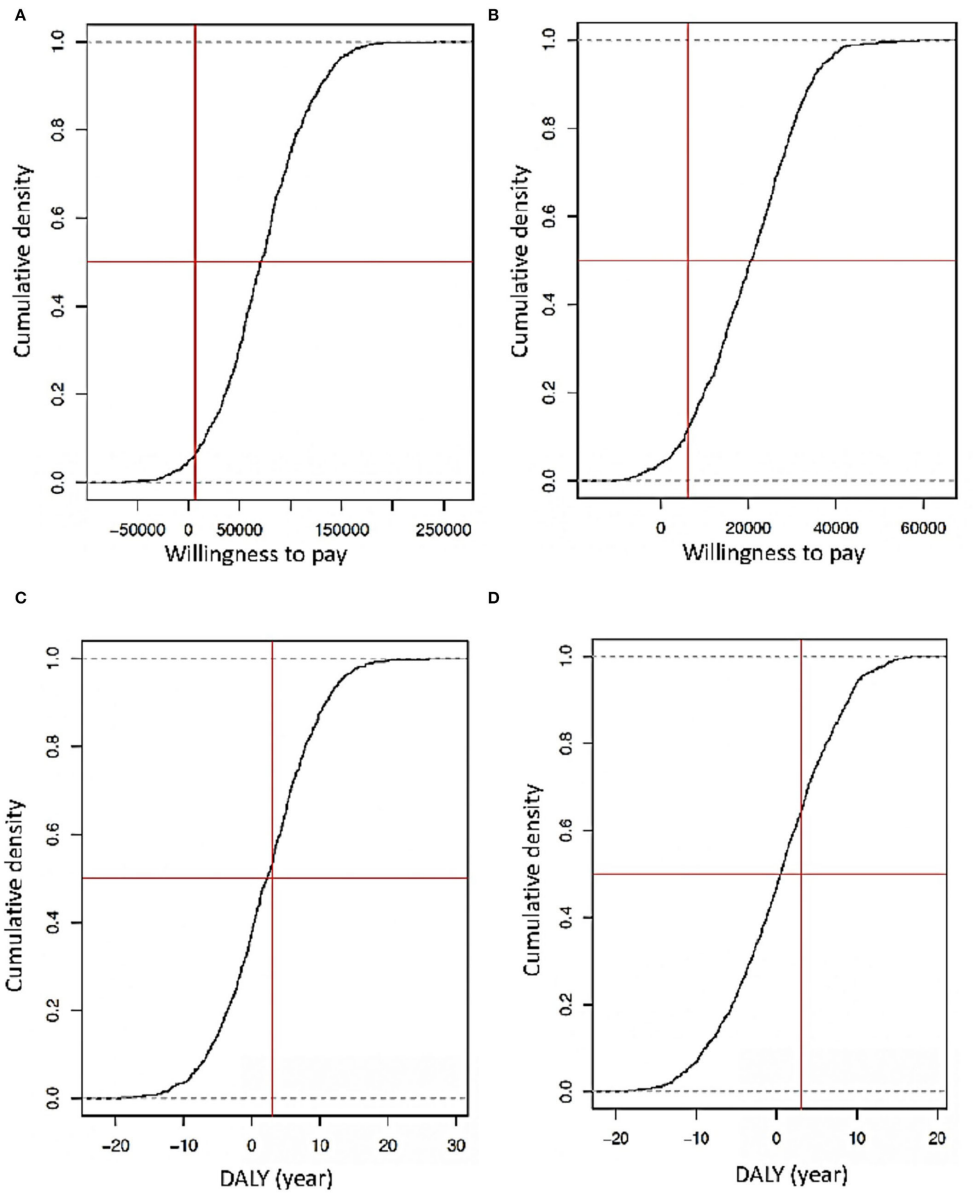
**(A)** Simulation of DALY's years' loss or gain using deterministic data for the combination of ART and PrEP. **(B)** Simulations of DALY's years gained or loss using stochastic data for the combination of ART and PrEP (the Y-axis is the probability that an expending would be accepted and forming part of the implementation strategy, X-axis represents how much a government can spend on a plan according to its GDP, represented by a vertical red line, and the horizontal red line is the logistic regression threshold of 50%). **(C)** Simulations of DALY's years gained or loss using deterministic data for ART and PrEP. **(D)** Simulations of DALY's years gained or loss using stochastic data for the combination of ART and PrEP (* the Y-axis is the probability of loss or gain years of life due to an implemented strategy. The vertical red line represents the standard DALYs lost by a later diagnosis, and the horizontal red line is the logistic regression threshold of 50%).

With PSA for ART and PrEP, and if these interventions are separated, their costs are high. For ART, using stochastic and deterministic data showed an investment between 20,000 and 50,000 USD per person in the simulated population, but a national strategy budget is required. In contrast, for PrEP, the value is less (2,000–5,000 USD per person in the simulation) and is under the GDP threshold (Figure 7). Considering DALYs with the same type of data, if the budget reduces more than 50% to ART, it will generate a loss of 10–12 years of healthy life. In PrEP, the loss could be between 6 and 8 years of healthy life (Figure 8). Finally, the strategy of using both ART and PrEP with stochastic and deterministic data would require an investment of 16,000–50,000 USD per person in the simulated population. This would also be under the GDP's threshold (Figures 9A,B). For DALYs, this combination strategy and the decrease of 50% in the local budget would lead to a loss of 4–6 years of healthy life (Figures 9C,D).

## Discussion

HIV is categorized as a chronic transmissible disease, which is still an issue for public health, generating a significant expense due to a delay in diagnosis and treatment. Although ART and optimal diagnosis strategies have reduced its morbimortality, there is no definitive cure or eradication of the virus from their hosts. Thus, local and international authorities should reinforce the prevention and early detection of HIV infections. According to WHO and MSP in 2019, Ecuador showed a decrease in HIV mortality (<4.55/10,000 inhabitants) and its incidence (<2.2 cases/1,000 inhabitants), indicating that the interventions implemented by the national authorities have been effective (8). However, they are not enough for controlling the HIV epidemic as some reevaluations and new implementations are required (13).

The cost–utility analyses (CUA) are highly significant since they provide essential information and should be considered during the establishment, restructuring, implementation, or maintenance of the strategy as a control and prevention mechanism in case of an event. There is a misconception that knowing the representative cost of any plan is the only factor required in formulating health budgets. A CUA helps to determine whether a country could invest in a specific strategy to improve the overall situation and to choose the best response against a health event, based on a GDP's threshold and the calculation of expenses with a correct budget in a non-arbitrary way.

CUA is also thoroughly recommended in diseases like HIV, where diagnosis, prevention, and treatment need a constant investment that should be continuously monitored or modified according to results. For example, an early HIV diagnosis depends entirely on a regular screening among critical populations to reach an effective infection control, but this only could be possible with well-conducted financial investment. During 2012–2020, the WHO spent 40 million dollars only on HIV/AIDS diagnosis (30). Countries like the United States in 2008 pointed out that an investment of 3 dollars per person was used for rapid HIV tests annually (31). In 2017, Ecuador invested 49% of its health budget only for HIV prevention. Nonetheless, the exact percentage used only in screening is still unknown (8).

The US Center for Control and Prevention of Diseases (CDC) suggests that an extra effort should be made on screening the population who maintains an active sex life (people between 13 and 64 years old) with or without traditional risk behaviors (sex workers, MSM, etc.). All of this is a part of continuous surveillance and routine medical checks (32). Ecuador has included this policy in its new HIV clinical practice guideline from 2019, along with the idea of gradual implementation. On the contrary, Spain served as a reference for successful implementation of this early screening for the target population mentioned before, as in 2018 reached a detection of 90% HIV-infected population (33) with a high incidence among MSM. Also, Murray et al. (34) mentioned that using this strategy reduces ART's cost significantly and the expenditure in morbimortality and healthy years gain (21).

With all these in mind, we applied a CUA and PSA using nationally available data to appreciate if a strategy where all the sex-active population could be screened is cost-beneficial. According to our results, the ICER showed that the mentioned strategy (using stochastic and deterministic data) is cost–utility. An increase of 15–35% of additional investment from the HIV national budget keeps being under our national spending capacity and the GDP's threshold. Thus, we will generate source savings in the medium-term future (the negative sign of ICER) (35, 36).

Moreover, we considered in the analysis DALY's values that demonstrate an early screening remains cost-beneficial by preventing the infection than an investment in ART for the long term. The same was concluded by Cylus et al. (37). Although a gain of healthy years for early HIV identification is observed, it also depends on each subject's age at the time of diagnosis, since people more than 70 years old lose 0.3 healthy years without ART, and people between 15 and 49 years old with the same condition can lose 21 DALY's years. So the profit of healthy years for an early diagnosis decrease as age increases (38, 39).

We simulated a scenario where 50% of the typically assigned budget to HIV diagnosis was reduced without any CUA analysis. It demonstrated a loss of years of healthy life (5 years/DALY's), meaning that HIV carriers, with this budget reduction, would lose up to 5 years of life, revealing the importance of continuing and improving a diagnosis strategy of this infection. Furthermore, with a combination of screening of the sexually active population and the early identification of cases and prompt use of ART, but 7% of budget reduction, a loss of 20 years/DALY's in the HIV population could occur (35, 40).

Ecuador health investment compared to its GDP increased in 2012 to 2.5% compared to only 0.6% in 2000. This only demonstrates that a slight decrease in health budget could be one explanation for the increase in HIV cases during those years and, in consequence, less healthy years and a 2.5 DALY's% of health budget (35, 40). Ecuador invests at least 40% of its HIV budget in ART than in prevention, but, in future, this strategy will generate more costs than source savings. The same pattern can be found in other countries.

The PAHO and other organizations have mentioned that a reduction in HIV/AIDS incidence was achieved by ART coverage (not an early diagnosis) (8, 41). However, in 2013, HIV/AIDS ranked number 10 in the causes of loss of years of healthy life (DALY's), and in 2017, it reached number two, showing that even though the use of ART and the adopted measurements are useful, they prove not to be enough to get an optimal control and management of HIV/AIDS. Also, ART may cause a loss of 1.4 healthy years by following our model results where drug treatment generates a loss of 2 to 2.5 of healthy years. It is important to emphasize that this loss does not support the idea that an HIV carrier should not receive treatment (21, 38), because it generates a loss of 60 years of health.

PrEP has been gaining considerable ground in the last decades, showing it is a good option for HIV prevention, especially at high-risk groups, but not on a large scale. A successful follow-up and control have been reached in small groups and specific locations. On the contrary, on an upper rate, it has been demonstrated that it does not work well if the cost increases and if it is not cost-beneficial (42, 43). In our analysis, we observed the same pattern in the simulations. PrEP had a lower cost compared to the costs generated by ART-like treatment. But considering that PrEP is not used in seropositive patients, and it is exclusive given to populations at high risk to acquire HIV, applying the early screening strategy (proposed above) to all sexually active people is not feasible, due to its high costs. Through ICER calculation, we exhibited that PrEP is less expensive than ART and with fewer DALYs dropped.

Nevertheless, the PSA was close to exceeding the amount (closely reaching a high cost and low-efficiency position). Comparing the PSA between ART and PrEP, regarding DALY's, a difference of 3–5 DALY's years was observed. This means that if PrEP is used among high-risk populations, a boost in healthy years could be reached, similar to what was observed in previous studies, where the loss of healthy years with ART and PrEP are 7 and 5 years, respectively (44).

We estimated the scenario at which early screening and use of ART occurred and observed that this combination could reduce up to three DALY's years. In addition, the ICER's consolidation is more profitable, following the WHO report, increasing healthy life years and diminishing costs. All of this, along with a constant investment, allow savings and infection control (45). On the contrary, if ART is used late, even though the diagnosis was early, a loss of nine DALYs could have experimented. The fusion of PrEP and an early diagnosis can generate a loss of up to two DALYs years, but if PrEP is not used in high-risk population and only early diagnosis is used, a decrease of eight DALYs years could be expected. Following HIV/AIDS reports regarding global economic issues, the coverage of pharmacological HIV treatments has decreased, leading to loss of 5 to 10 years/DALY's due to imperfect execution of strategies or lack of access to ART (38). Thus, there are cases in countries where PrEP or ART is intermittent without a timely diagnosis, provoking what a phenomenon called “islands of infection” where the accumulation of new cases is observed, and a spread of the HIV infection occurs (43, 46).

It is crucial to mention the weaknesses that this type of model has, which is calculated by GPD's thresholds in each country. According to the WHO, during the 90s, calculating a general threshold was complicated. For this reason, the GDP per country was proposed as a reference (37). Our models showed that according to the national context, it is cost-beneficial to apply an early screening. Even by considering our GDP's threshold, we should invest 49% of the national income, exclusively on the diagnosis and treatment of HIV. With this in mind, a solution could be to follow the English Health System that calculates a threshold based on the comparison of the total health gained by prevention against purchasing power parity of a new treatment. Usually used as an index, it portrays the real value to pay per habitant, for each government to be able to increase the population's healthy years. For England in 2013, this value was 4,260 USD (37). In Ecuador, it represents more than 80% of GDP, but we could find a real value with an accurate calculation.

## Conclusion

Finally, the CUA demonstrates that an early diagnosis in a sexually active population is cost-beneficial. This, combined with ART or PrEP, is ideal to acquire more years of healthy life. If ART and PrEP were considered, this would be cost-beneficial but only aimed at the high-risk population; otherwise, its use is not justified. Maintaining a fixed budget for the HIV / AIDS programs, a thing that does not vary according to the government in office, will allow resource savings due to the decrease of DALY's. A thing that in future will allow the ART and PrEP costs are redefined. This may generate savings and improve the strategy toward early diagnosis, reach 90–100% ART coverage, meet the WHO target, and achieve a 15–30% budget savings with this strategy.

## Data Availability Statement

The raw data supporting the conclusions of this article will be made available by the authors, without undue reservation.

## Author Contributions

PQ-A, PE, and ET conceptualized the study. SO, IH, and AH provided technical input for the methodology and analysis. PQ-A and PE collected and analyzed data. SO, IH, AH, and ET reviewed the analysis and validated the results. All authors contributed to writing the initial draft, reviewed, and approved the final version.

## Conflict of Interest

The authors declare that the research was conducted in the absence of any commercial or financial relationships that could be construed as a potential conflict of interest.

## Publisher's Note

All claims expressed in this article are solely those of the authors and do not necessarily represent those of their affiliated organizations, or those of the publisher, the editors and the reviewers. Any product that may be evaluated in this article, or claim that may be made by its manufacturer, is not guaranteed or endorsed by the publisher.
